# Cell-Free Wharton’s Jelly-Derived MSC Secretome in a Polymeric Carrier for Refractory Chronic Wounds: Two Cases

**DOI:** 10.3390/bioengineering13091077

**Published:** 2026-09-16

**Authors:** John A. Dangerfield, Arkar W. Maung, Poovasit Klinoubol

**Affiliations:** 1 Miskawaan Health Group, 2415/4 New Phetchaburi Road, Bangkapi, Bangkok 10310, Thailand; 2 Foot Clinic and Wound Care, Vimut-Theptarin Hospital, Bangkok 10110, Thailand

**Keywords:** mesenchymal stromal cell secretome, cell-free therapy, Wharton’s jelly, thermoresponsive carrier, drug delivery, regenerative medicine, chronic wounds, diabetic foot ulcer, peripheral arterial disease, wound closure

## Abstract

Background: Chronic wounds that fail to heal despite debridement and advanced care are an unmet clinical need, particularly in elderly patients and those with peripheral arterial disease, for whom the alternatives are often indefinite maintenance or amputation. The mesenchymal stromal cell (MSC) secretome is pro-reparative in preclinical models, but human evidence is concentrated on shorter-duration diabetic foot ulcers and has usually reported area reduction rather than complete closure. Methods: We describe two patients with long-standing, treatment-refractory wounds treated with a cell-free Wharton’s jelly-derived MSC secretome delivered in a thermoresponsive polymeric carrier that gels at the wound bed, applied every three days alongside standard care. Results: The cases comprise the following: Case 1, a 95-year-old woman with peripheral arterial disease and a two-year ankle ulcer refractory to multiple advanced products; and Case 2, a 69-year-old man with a one-year neuro-ischemic diabetic foot ulcer persisting after amputation and repeated debridement. Both achieved complete closure at approximately 27 and 28 weeks, respectively, from baseline wound areas of approximately 7.5 cm^2^ (Case 1) and 12 cm^2^ (Case 2), using approximately 20 mL of preparation each. Closure was sustained at the latest follow-up (27 March 2026, Case 1; 21 June 2026, Case 2). Treatment was well tolerated; Case 2 had an intercurrent infection requiring a brief pause. Conclusions: A two-patient series cannot establish efficacy, but complete closure in wounds this refractory supports controlled evaluation of this cell-free platform, with complete closure as the primary endpoint.

## 1. Introduction

Chronic wounds are among the most stubbornly unsolved problems in clinical medicine. Defined by their failure to progress through the ordered phases of repair, they persist for months or years, and their principal forms—diabetic foot ulcers, vascular ulcers, and pressure injuries—share a common endpoint despite differing origins: a wound bed locked in a self-sustaining state of inflammation, impaired angiogenesis, and arrested re-epithelialization [1]. The clinical consequences are severe, extending to infection, hospitalization, and limb loss, and the economic burden is measured in tens of billions of dollars annually in the United States alone [2,3]. An aging population with rising rates of diabetes and peripheral arterial disease ensures that this burden continues to grow.

Development of therapeutic intervention has been incremental rather than transformative. Debridement, offloading, moisture balance, and infection control remain the foundation of care, and the advanced modalities layered on top—bioengineered skin substitutes, extracellular matrix scaffolds, topical oxygen, negative-pressure therapy, and growth factor preparations—improve outcomes for some patients but leave a substantial minority unhealed [4]. This refractory group is clinically distinct: for wounds that remain open after every available intervention has been tried, in patients whose age or vascular status makes surgical reconstruction unattractive or impossible, the realistic alternatives become indefinite maintenance or amputation [5].

Mesenchymal stromal cells (MSCs) have long been proposed as a way out of this impasse, and it is now widely accepted that their reparative influence is exerted largely through paracrine signaling rather than engraftment and differentiation [6]. Cells transplanted into a hostile wound bed survive poorly, yet still accelerate healing—an observation that has shifted attention decisively from the cells themselves to what they secrete [7]. The MSC secretome, a complex mixture of growth factors, chemokines, matrix modulators and extracellular vesicles (EVs), reproduces the parent cells’ activity while dispensing with many challenges associated with their handling [8,9]. As a cell-free material, it can, in principle, be characterized, stored, and standardized, and it carries no risk of uncontrolled proliferation or ectopic differentiation; such preparations have accordingly been proposed to reduce the cold-chain infrastructure and specialist handling that have kept living-cell therapies confined to well-resourced centers [8,9]. Among available sources, umbilical cord Wharton’s jelly is particularly attractive: the tissue is obtained non-invasively from routinely discarded material which can be ethically donated, and the cells derived from it are developmentally young, expand robustly, and secrete a factor profile weighted towards angiogenesis and matrix remodeling [10].

The preclinical evidence is now extensive and consistent. Across cell-based assays, three-dimensional skin equivalents, and rodent wound models, MSC-derived secretome and EV fractions, including exosomes, accelerate re-epithelialization, promote angiogenesis, recruit fibroblasts, and hasten resolution of inflammation [11,12,13]. Translation into patients, however, remains at an early stage. The available human evidence, including randomized studies of Wharton’s jelly-derived EVs and early-phase trials of conditioned media, has focused almost exclusively on diabetic foot ulcers, typically of relatively short duration, and has most often reported percentage reduction in wound area rather than complete closure [14,15]. Purified EV fractions have dominated, despite the cost and complexity of their manufacture and evidence that the broader secretome retains the full complement of soluble mediators [16]. Little has been published on the wounds that matter most clinically: those open for a year or more, already refractory to surgery and to multiple advanced products, and in patients with significant arterial disease or at the extremes of age. A further, often neglected obstacle is delivery. A low-viscosity liquid preparation (e.g., culture medium) applied to an exuding, irregular wound bed is quickly lost, and sustained contact between the secretome and the wound surface is a prerequisite for any significant biological effect.

Conventional wound-delivery formats address this only partially. Simple dressings and aqueous or semi-solid vehicles either wash out of an exuding, contoured wound bed or must be reapplied so frequently that sustained exposure is difficult to maintain, while preformed hydrogel sheets conform poorly to irregular cavities and undermined margins [17]. Thermoresponsive (in situ gelling) polymers offer a way around this trade-off: applied cold as a low-viscosity liquid, they flow into and coat an irregular wound surface and then gel at skin and body temperature to form a conformal depot that resists displacement and holds a payload in prolonged contact with the tissue [18]. Poloxamer-based thermoresponsive carriers are among the best-characterized systems of this class and have an established record as biocompatible vehicles for topical and wound applications [18]. Coupling a whole-secretome biologic to such a carrier—the approach whose preclinical development we described previously [11]—is a rational strategy to convert a transiently applied liquid into a retained, wound-contacting reservoir, and provides the design rationale for the platform reported here.

Here, we address these gaps directly. We report two patients—one aged 95 with a post-traumatic ankle ulcer complicated by peripheral arterial disease and open for two years despite a sequence of advanced wound care products, the other aged 69 with a neuro-ischemic diabetic foot ulcer persisting for a year after amputation and repeated debridement—in whom a cell-free Wharton’s jelly-derived MSC secretome, formulated in a thermoresponsive polymeric carrier to maintain contact with the wound bed, was applied as adjunctive compassionate treatment under the care of the treating physician. Both wounds, having failed all prior therapy, went on to close completely during the treatment period, without treatment-related adverse events. We present these two cases as an initial clinical proof-of-concept for this engineered, cell-free platform (a whole-secretome biologic coupled to a thermoresponsive delivery carrier) in wounds that had exhausted the available standard of care.

## 2. Materials and Methods

### 2.1. Study Design and Setting

This is a retrospective description of two patients with chronic, non-healing wounds treated with a cell-free mesenchymal stromal cell secretome preparation, delivered in a thermoresponsive polymeric carrier, as individualized compassionate clinical management. Both patients were managed by the treating physician (P.K.), a specialist in podiatric medicine and surgery, at the Foot Clinic and Wound Care service, Vimut-Theptarin Hospital, Bangkok, Thailand. In each case, the secretome preparation was offered as an adjunct to continued standard of care after the wound had failed to progress under conventional treatment; no randomization, control group, or research-specific procedures were involved, and all clinical decisions were made by the treating physician in the patient’s interest. Data were extracted retrospectively from de-identified clinical records and serial clinical photographs. The clinical cases are reported in accordance with the CARE (CAse REport) reporting guidelines [19].

### 2.2. Secretome Preparation and Quality Control

The material used was a cell-free mesenchymal stromal cell secretome: a concentrated preparation derived from the serum-free conditioned medium of human umbilical cord Wharton’s jelly-derived mesenchymal stromal cells, produced by prolonged, high-density culture. The harvested conditioned medium was fractionated and concentrated 50-fold by ultrafiltration, with only the retentate retained, as previously described [11]. Throughout this manuscript, “secretome” refers to this concentrated retentate preparation; “conditioned medium” refers to the unconcentrated culture supernatant from which it is derived; and “extracellular vesicles (EVs)” denotes the vesicular fraction retained within the secretome, of which exosomes are one subtype. For topical application, the minimally processed and concentrated secretome was incorporated into a poloxamer-based thermoresponsive polymeric carrier—the same class of carrier prepared from the same poloxamer reagent and used in the foundational preclinical work [11]. In aqueous solution, the carrier is a free-flowing liquid at low temperature, permitting mixing, loading and delivery, and undergoes a reversible sol–gel transition as it warms towards skin and body temperature to form a soft in situ gel, thereby retaining the secretome in sustained contact with the wound bed following application; its thermoreversible behavior and formulation have been described and characterized previously [11]. Poloxamer is a well-established, biocompatible polymeric carrier widely used as a pharmaceutical excipient and incorporated into approved topical and wound-care products [18]. The reagent used here was of research (cell-culture) grade rather than a pharmacopeial excipient lot, and the finished secretome–carrier preparation was applied on a compassionate, named-patient basis rather than under a marketing authorization for wound management. The specific carrier composition and concentration are proprietary to the manufacturer and are not disclosed here; the general formulation approach and the concentrations evaluated during preclinical development are reported in the foundational study [11].

The secretome used for both patients was derived from a single master cell bank (one umbilical cord donor). The finished secretome–carrier preparation was supplied as two lots produced to identical materials, protocols and quality-control specifications; each patient received approximately equal quantities from both lots, and the consistent healing trajectory across the treatment period gave no indication of lot-to-lot variation in performance.

The preparation used in these cases was of research grade and was supplied as a finished product by Celligenics Pte Ltd, Singapore; its individual formulation components are proprietary to the manufacturer and are not separately disclosed. All starting materials, culture media, and carrier components were obtained as sterile, certified reagents supplied with certificates of analysis. Sterility of each batch was verified in-house prior to clinical use: test material was incubated in sterile culture medium (DMEM; Gibco, Thermo Fisher Scientific, Waltham, MA, USA) for 72 h and examined microscopically for microbial growth, assessed as the appearance of particulates or turbidity, alongside a negative (medium-only) control. Absence of detectable particulates at 72 h was required for release, and any detectable particulates rendered the batch unsuitable for use. This screening was applied to the secretome, the carrier, and the final combined preparation. No positive growth control was included, and no compendial sterility or bacterial endotoxin testing was performed; endotoxin was not separately assayed, on the basis that all inputs were supplied sterile and no microbial growth was detected at any stage. These limitations are addressed in Section 4.3.

### 2.3. Treatment Protocol

Following debridement, the secretome–carrier preparation was applied at approximately 3-day intervals, timed to the routine dressing-change schedule and to the expected in situ residence of the gelled carrier, so as to maintain sustained exposure with minimal disturbance of the wound bed. It was dripped from above as a thin covering layer, without the syringe tip contacting the wound. The per-application volume could not be precisely measured but was estimated at up to ~0.8 mL (≈16 drops) on the larger, earlier wounds, falling to ~0.1–0.2 mL (2–4 drops) as the wounds approached closure, and was determined by wound size at the treating physician’s discretion. Approximately 20 mL was used per wound over the full course, supplied in re-sealable 5 mL screw-cap syringes (BD; Becton, Dickinson and Company, Franklin Lakes, NJ, USA). The carrier’s slight viscosity in its liquid state facilitated application relative to an aqueous-conditioned medium, and a non-absorbent secondary dressing was applied over the preparation to protect the applied layer and prolong its retention at the wound surface. Standard wound care continued throughout, comprising periodic debridement, moisture-balancing dressings, application of a barrier preparation to the periwound skin, and offloading or pressure management as clinically appropriate. Treatment continued until complete wound closure. The number of applications and total treatment duration for each patient are given in Section 3 and Table 1.

### 2.4. Wound Assessment and Outcomes

Wounds were assessed at each clinical visit by the treating physician. Wound dimensions (length × width × depth, cm) were recorded using a disposable paper wound ruler (unbranded), and wound-bed characteristics (granulation, slough or necrosis, exudate, and epithelialization) were documented and supported by serial clinical photography. The primary outcome was complete wound closure, defined as full re-epithelialization of the wound surface with no residual open area and no drainage, as determined by the treating physician [20]. Given the descriptive nature of a two-patient series, no formal statistical analysis was performed.

### 2.5. Photographic Documentation and Figure Preparation

Serial clinical photographs were obtained during routine wound care by the treating physician using standard digital photography. Images were acquired without standardized lighting, fixed camera distance, or in-frame scale reference, and are therefore presented as qualitative documentation of wound progression; all quantitative wound dimensions reported in this manuscript were obtained by direct clinical measurement (Section 2.4) rather than from photographs.

**Figure 1:**
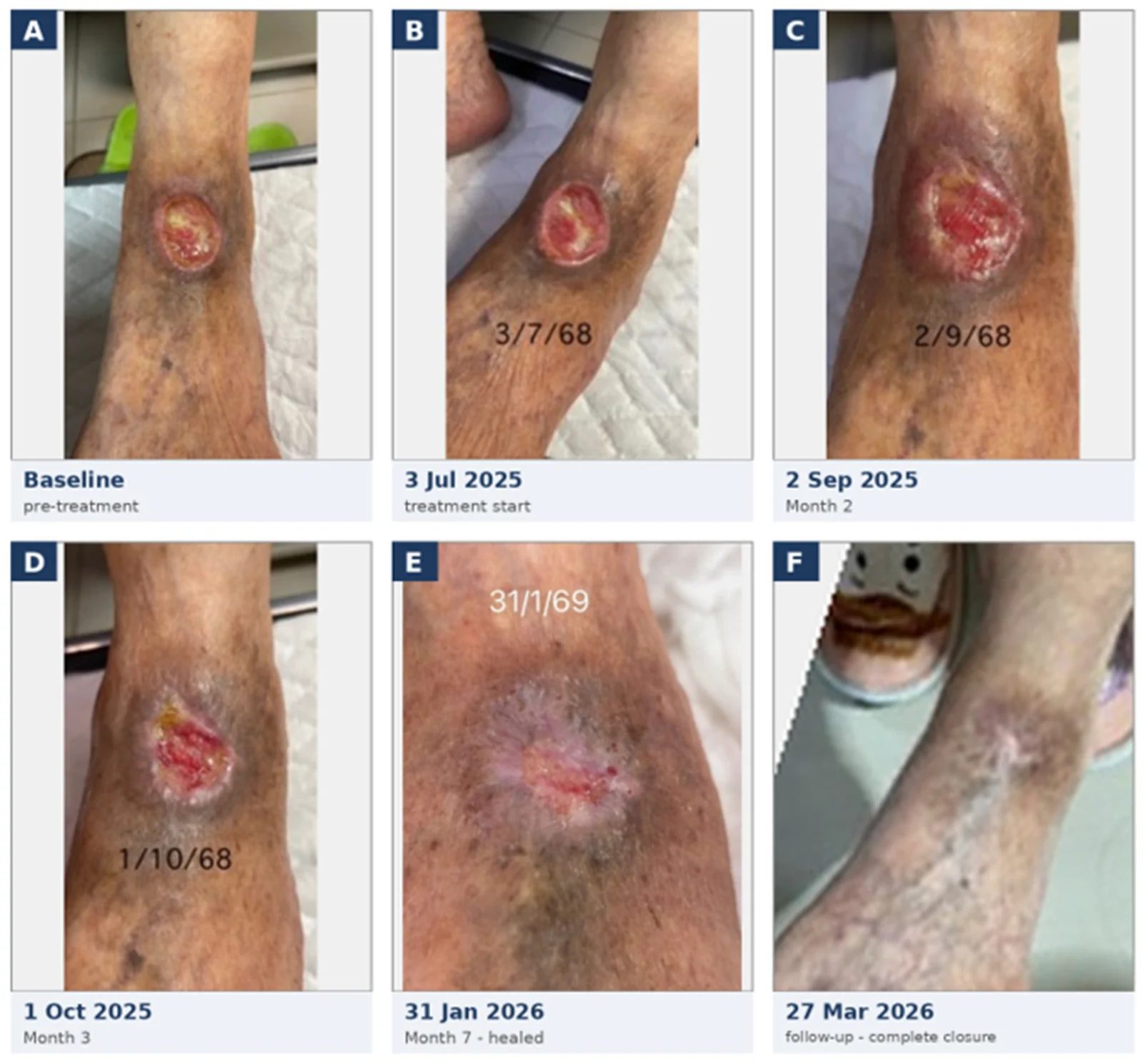
Progressive healing of a refractory peripheral arterial disease (PAD)-associated ulcer of the left ankle in a 95-year-old female, treated topically with cell-free Wharton’s jelly-derived mesenchymal stromal cell secretome in a thermoresponsive polymeric carrier. (**A**) Baseline, pre-treatment: chronic full-thickness ulcer with a sloughy, poorly granulating bed. (**B**) 3 July 2025, at commencement of treatment. (**C**) 2 September 2025 (~2 months): established granulation with margin contraction. (**D**) 1 October 2025 (~3 months): marked area reduction with healthy granulation. (**E**) 31 January 2026: complete closure sustained at follow-up (closure first documented 8 January 2026). (**F**) 27 March 2026: further follow-up confirming durable, complete closure. Images are de-identified and cropped to the wound; panel F has been reframed and rotated to approximate the viewing distance of the other panels (wound dimensions in Table 1).

**Figure 2:**
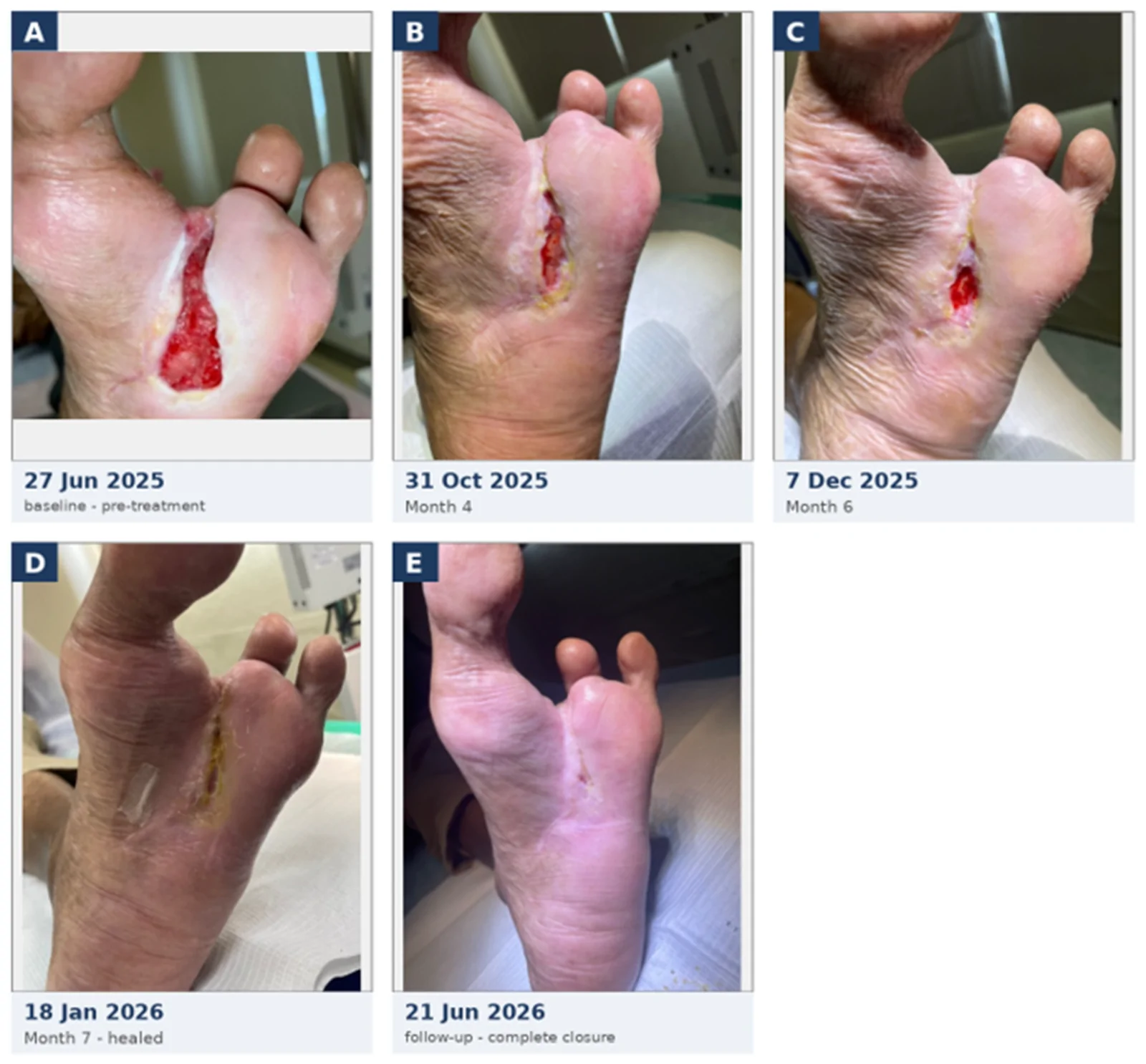
Progressive healing of a refractory neuro-ischemic diabetic foot ulcer with peripheral arterial disease (PAD) at the plantar forefoot in a 69-year-old male, treated topically with cell-free Wharton’s jelly-derived mesenchymal stromal cell secretome in a thermoresponsive polymeric carrier. The wound had remained refractory for approximately one year despite prior amputation, repeated surgical debridement, intravenous antibiotics and advanced local wound care. (**A**) 27 June 2025, prior to treatment: open, debrided plantar forefoot wound with a fully granulating base; secretome treatment commenced on 4 July 2025. (**B**) 31 October 2025 (Month 4): granulation with margin contraction and resolution of the full-thickness undermining. (**C**) 7 December 2025 (Month 6): substantial area reduction with healthy granulation and circumferential epithelialization. (**D**) 18 January 2026 (Month 7): complete closure, with thin epithelial cover over the wound surface. (**E**) 21 June 2026: follow-up confirming durable, complete closure. Images are de-identified and cropped to the wound (wound dimensions in Table 1).

Figure 1 and Figure 2 were assembled from unaltered original photographs. An AI assistant (Claude Opus 4.8; Anthropic, San Francisco, CA, USA; https://www.anthropic.com, accessed on 20 August 2026) was used to support panel assembly, cropping, framing and the addition of panel letters and date labels. In Figure 1, panel F was rotated and cropped to approximate the viewing distance of the other panels. No image content was generated, enhanced, retouched or otherwise modified, and no adjustments were made to color, contrast, or wound appearance. All panel assignments and dates were verified against the source clinical records by the treating physician (P.K.).

## 3. Results

### 3.1. Case 1: Refractory Chronic Ankle Ulcer in a 95-Year-Old Patient with Peripheral Arterial Disease

A 95-year-old woman with a background of peripheral arterial disease, maintained on clopidogrel and low-dose aspirin for secondary prevention of thrombotic events, presented with a chronic ulcer on the anterior aspect of the left ankle. The wound originated in October 2023 after the patient scratched an insect bite, which subsequently became infected, and had remained open for approximately two years. Prior management had included surgical debridement and a succession of advanced wound care modalities—topical oxygen therapy, Granulox (topical hemoglobin spray), a protease-modulating dressing, Endoform (ovine forestomach extracellular-matrix dressing), and Kerecis (omega-3 acellular fish-skin matrix). The wound had initially approached closure but subsequently deteriorated over the preceding seven months despite consistent standard of care, and was therefore considered refractory to available treatments.

The ulcer had reached a maximal recorded size of 3.8 × 4.0 × 0.2 cm. At baseline assessment on 31 May 2025, immediately prior to treatment, it measured 2.5 × 3.0 × 0.2 cm (an approximate wound area of 7.5 cm^2^, calculated as the length × width product of ruler measurements rather than by planimetry). The wound bed showed a mixed fibrotic and granulating base in an approximate 70:30 ratio, with a rolled wound border and hyperpigmented surrounding skin. There was no probing to bone, no undermining, and no tunneling. Doppler assessment of the left leg demonstrated strong but monophasic waveforms at the dorsalis pedis and posterior tibial arteries, with a biphasic signal at the popliteal artery, consistent with peripheral arterial disease.

Treatment with the secretome preparation began on 3 July 2025 and was applied every 3 days. A total of four syringes (approximately 20 mL) were used over the treatment course. Adjunctive care comprised weekly debridement for the first three months, thereafter reduced to bi-weekly debridement, performed carefully so as to avoid bleeding, together with application of a skin barrier preparation around the wound margin to protect the patient’s fragile periwound skin.

The wound responded progressively. By week 5, it had reduced to 2.0 × 2.0 × 0.1 cm (~4.0 cm^2^, a ~47% reduction from baseline), with a softer granulating base covering more than 90% of the wound surface and resolution of the rolled border. By week 9, the wound measured 1.3 × 1.8 × 0.1 cm (~2.3 cm^2^, a ~69% reduction), with near-complete granulation coverage and epithelialization advancing from the 12 to 9 o’clock positions. At week 14, the wound measured 1.2 × 1.5 × 0.1 cm (~1.8 cm^2^, a ~76% reduction) with a fully granulating base and circumferential epithelialization.

Complete wound closure, with a thin epithelial cover over the entire wound surface, was documented on 8 January 2026. Sustained closure was confirmed at follow-up assessments on 31 January 2026 and 27 March 2026, with no evidence of recurrence or breakdown (Figure 1). Total time to complete closure was approximately 27 weeks. No adverse events, wound infection, or treatment-related complications occurred during the treatment period.

### 3.2. Case 2: Refractory Neuro-Ischemic Diabetic Foot Ulcer in a 69-Year-Old Patient

A 69-year-old man with type 2 diabetes mellitus complicated by peripheral neuropathy and peripheral arterial disease, maintained on low-dose aspirin, presented with a chronic neuro-ischemic diabetic foot ulcer on the plantar aspect of the forefoot, a weight-bearing location characteristically slow to heal. His diabetes was of more than 20 years’ duration, managed with oral hypoglycemic agents and dietary control. He had severe sensory neuropathy, with absent protective sensation on 10 g monofilament testing and impaired vibration perception on 128 Hz tuning-fork examination bilaterally and characteristically bore weight on the plantar ulcer without pain. The wound had been present since June 2024, approximately one year prior to treatment, and had persisted despite prior partial amputation of the second ray and third toe, repeated surgical debridement, a prolonged course of intravenous antibiotics, and advanced local wound care—initially Granulox applied topically and subsequently a protease-modulating collagen dressing with silver antimicrobial foam (Allevyn Ag)—which had resulted in near-complete granulation of the wound bed but not closure. PolyMem foam, a hydrophilic polyurethane matrix dressing, was subsequently applied to facilitate wound closure; however, complete closure was not achieved at that time.

The ulcer had reached a maximal recorded size of 3.0 × 6.5 × 0.7 cm. At baseline assessment on 27 June 2025, it measured 2.0 × 6.0 × 0.3 cm (an approximate wound area of 12 cm^2^), with the wound bed fully covered by granulation tissue (100%). Full-thickness undermining, previously extending approximately 2 cm (1 to 3 o’clock), had reduced to approximately 1 cm at baseline.

Treatment with the secretome preparation began on 4 July 2025 and was administered every 3 days, with the frequency reduced to weekly during the final month of treatment. In total, approximately four syringes (20 mL) were used. Adjunctive wound care consisted of bi-weekly debridement, cleansing with polyhexamethylene biguanide (PHMB) solution, intermittent offloading with custom-cut adhesive felt, and application of a non-adherent contact layer (UrgoTul) beneath a foam dressing with a second non-adherent wound-contact layer (Allevyn). During the second month of treatment, the wound became infected (first noted on 21 September 2025), and secretome application was temporarily suspended for approximately four weeks. Wound cultures grew *Stenotrophomonas maltophilia* and *Staphylococcus aureus*. Empirical clindamycin plus ciprofloxacin was given for one week, followed by culture-directed levofloxacin, to which both isolates were susceptible. During this interval, the dressing regimen was changed to a daily topical antibiotic ointment. Secretome treatment was resumed on 19 October 2025, following clinical resolution of the infection. The infection was not considered related to the preparation: *S. maltophilia* and *S. aureus* are common pathogens of chronic diabetic foot ulcers at weight-bearing sites, the preparation is acellular, no local hypersensitivity or reaction to it was observed at any time, and healing resumed and proceeded to complete closure once the same preparation was reintroduced.

Progressive contraction, granulation, and epithelialization were observed. By Month 4 (31 October 2025), the wound measured 0.5 × 2.3 × 0.1 cm (~1.2 cm^2^, a ~90% reduction from baseline), with a fully granulating base (100%), resolution of the full-thickness undermining, resolution of the epibole, and epithelialization advancing circumferentially. By Month 6 (7 December 2025), it measured 0.5 × 1.5 × 0.1 cm (~0.8 cm^2^, a ~94% reduction), with firm central granulation and epithelialization around the entire wound margin.

Complete wound closure, with thin epithelial cover, was documented on 18 January 2026 (Month 7; Figure 2), giving a total time to complete closure of approximately 28 weeks from treatment initiation. Closure was sustained at follow-up on 21 June 2026, approximately five months later, with the healed skin closely resembling the surrounding native skin and only a small residual callus. Aside from the second-month wound infection described above, no adverse events, local reactions, or treatment-related complications occurred.

### 3.3. Summary of Outcomes

Both wounds achieved complete closure. In both cases, the wounds had been present for one to two years and had failed to heal despite surgical debridement and, in Case 1, multiple advanced wound care products; in Case 2, the wound persisted despite prior amputation and treatment for infection. In Case 2, glycemic control was reasonably well maintained during the treatment period, with HbA1c ranging from 6.7% to 7.1%, and the ankle-brachial index (ABI) was 0.71, for which no additional invasive vascular intervention was required. Over the treatment period, both wounds showed progressive reduction in area and depth, granulation, and circumferential epithelialization, culminating in complete closure, in patients aged 95 and 69 years with significant vascular comorbidity. In Case 2, treatment was interrupted for approximately four weeks by an intercurrent wound infection before healing resumed. Closure was achieved with modest total volumes of the preparation (approximately 20 mL per case). No local reactions to the preparation occurred; the only intercurrent complication was the Case 2 wound infection noted above. Baseline characteristics, treatment parameters, and outcomes are summarized in Table 1.

## 4. Discussion

Two chronic wounds, each open for a year or more and each having failed the treatments conventionally offered to such patients, closed completely after introduction of a cell-free Wharton’s jelly-derived MSC secretome delivered in a thermoresponsive polymeric carrier. Both had already exhausted advanced wound care—in Case 1, multiple advanced products in a 95-year-old with peripheral arterial disease; in Case 2, amputation, repeated debridement, and intravenous antibiotics in a 69-year-old with diabetes, neuropathy, and arterial disease. In both, complete re-epithelialization was achieved over the course of treatment following introduction of the secretome preparation; in Case 2, an intercurrent second-month infection required a brief interruption before healing resumed to closure.

### 4.1. Relation to Existing Evidence

The clinical literature on MSC-derived secretome in wound healing, though expanding rapidly, remains narrow in a specific way. Most human studies have enrolled diabetic foot ulcers of relatively short standing, and the primary endpoint has commonly been percentage reduction in wound area over a defined observation window rather than complete closure [14,15]. Percentage area reduction is a defensible surrogate for a trial, but it is not what determines whether a patient keeps a limb. The cases reported here differ in three respects that we believe are clinically meaningful. First, both wounds were of long duration and demonstrably refractory: each had already exhausted the interventions that would ordinarily be offered, which substantially weakens the argument that closure reflected the natural history of an otherwise treatable ulcer. Second, the endpoint achieved was complete closure rather than partial improvement. Third, the patients sit at the difficult end of the clinical spectrum, one of them 95 years old with arterial insufficiency, the other with combined neuropathy and ischemia at a mechanically unfavorable site; precisely the populations underrepresented in existing trials, in which age limits, comorbidity exclusions, and ulcer duration criteria tend to select for wounds more likely to heal. To the best of our knowledge, based on the literature reviewed, complete closure has not previously been reported in chronic wounds of this duration and refractoriness treated with a cell-free MSC secretome.

Our observations also bear on which secretome fraction should be used clinically. Much of the recent translational literature has favored purified EVs or exosomes on the premise that they contain the principal therapeutic cargo. Yet vesicle isolation is costly, technically demanding, and difficult to scale [21], and it discards the soluble fraction of growth factors, cytokines, chemokines, and matrix modulators, which are also biologically active. The material used here was the whole secretome, retaining both the EV fractions as well as the majority of soluble protein factors.

It should be noted that “whole secretome” does not mean unprocessed conditioned medium. The preparation is concentrated by ultrafiltration above a defined molecular-weight cut-off, and only the retentate is retained; the specific cut-off and the rationale for selection are proprietary to the manufacturer and are described in the foundational methods [11]. This step therefore functions as a coarse purification as well as concentration: low-molecular-weight species such as residual medium components, salts and other solutes, degraded peptides and nucleic acid fragments are largely removed with the permeate, while soluble protein factors and all EV classes are retained. Beyond simplifying the composition of the applied material, this passive depletion of small-molecule and degradation-derived species is a desirable feature for a preparation intended for repeated topical application to an open wound bed, in which the barrier to systemic exposure is compromised.

That two wounds of this severity closed under such a preparation is consistent with (though it cannot by itself confirm) a growing body of work suggesting that the whole secretome may be therapeutically sufficient for cutaneous repair, and that the soluble, non-EV fraction contributes to this activity in its own right rather than serving merely as a carrier and stabilizer for EVs [22,23]. Studies in which the EV fraction is selectively depleted from conditioned medium report attenuated but not abolished healing [23], the residual activity implicating the soluble mediators [24], while paracrine soluble factors alone recruit macrophages and endothelial lineage cells and accelerate wound closure [25]. The balance between EV and soluble contributions appears to be tissue-dependent; some injury models favor exosome-enriched fractions [26], but in skin, the available evidence supports retention of both compartments, which the whole-secretome preparation used here achieves. Whether this translates into superior clinical closure relative to a purified fraction remains a hypothesis these two cases cannot establish; a direct head-to-head comparison in a controlled setting would be required to test it.

### 4.2. Plausible Mechanisms

The biology offers a coherent account of what was observed. Chronic wounds are arrested rather than merely slow: the wound bed is held in a state of persistent M1-type inflammation, with excessive protease activity, degraded matrix, impaired angiogenesis, and fibroblasts and keratinocytes that are themselves functionally exhausted and poorly migratory [1]. A therapy that acts on a single node in this network—for example, supplying one growth factor or debriding necrotic tissue alone—tends to produce only transient improvement because the underlying pathology inhibits further progress. The MSC secretome acts across several of these nodes simultaneously [7]. Reported activities include promotion of endothelial proliferation and vessel formation [27], recruitment and activation of fibroblasts with subsequent collagen deposition [28], stimulation of keratinocyte proliferation and migration [28], extracellular matrix remodeling [7], and polarization of macrophages toward a reparative phenotype [29]. The clinical sequence we observed is consistent with this multi-target action: conversion of a fibrotic wound bed to healthy granulation tissue, resolution of the rolled wound edge in Case 1 (a hallmark of a stalled epidermal front) and subsequent circumferential epithelialization rather than closure from a single margin. None of these mechanisms was directly evaluated in the two patients reported here; they are drawn from prior preclinical and clinical work and are presented to frame the observed healing rather than to evidence a mechanism in these cases.

The polymeric carrier used for delivery merits comment. A secretome applied as a liquid to an exuding wound is rapidly diluted and lost, and biological activity presupposes sustained contact with the wound surface. A thermoresponsive carrier—liquid when cool, semi-solid at body temperature—allows the preparation to conform to an irregular wound bed, including undermined regions (as in Case 2), and to remain in place between dressing changes. Beyond retaining the secretome, the carrier confers the benefits long-recognized for occlusive and semi-occlusive dressings: it provides a physical barrier against contamination and maintains a moist wound environment, which supports keratinocyte migration and proliferation and is associated with more efficient healing than a dry wound bed [30]. Release from poloxamer gels of this type is well established: being physical, non-covalently crosslinked thermogels, they liberate entrapped payload predominantly by erosion and dissolution in an aqueous environment [18], progressively delivering the secretome to the wound bed. This was consistent with the clinical course: at each dressing change, the applied preparation had thinned relative to its appearance on application, without drying out, indicating in situ erosion and local release rather than absorption into the non-absorbent secondary dressing.

It is also plausible that sustained local delivery of the secretome contributes to the quality of the healed tissue; MSC secretome and conditioned medium have been reported to attenuate fibrosis and reduce hypertrophic and keloid scarring in preclinical models, including formulations combining conditioned medium with a hydrogel carrier [31,32,33]. Scar quality was not a formal endpoint in the present cases, and this remains a property observed in other settings rather than one these two cases establish. We therefore regard the delivery format as an integral part of the intervention rather than an inert accessory; the total volumes required to achieve closure were modest, at approximately 20 mL across the full treatment course for each wound.

### 4.3. Limitations

The constraints on what may be concluded from this report have been considered, and we want to state them plainly here. This is a retrospective series of two patients, without a control group, randomization, or blinding, and no causal inference may be claimed from such a design. Both patients continued to receive standard wound care throughout, including regular debridement and periwound skin protection, and the contribution of the secretome cannot be formally separated from that of the concurrent care, although it is highly relevant that both wounds had failed to heal under standard care, including debridement, for one to two years beforehand. In addition, because the secretome was delivered within a carrier, its biological contribution cannot be distinguished from any independent effect of the carrier itself, which, as an occlusive, moisture-retaining vehicle, may influence healing in its own right. Separating the contributions of the secretome, the carrier, and concurrent standard care would require a controlled design incorporating carrier-alone and secretome-plus-carrier arms. Spontaneous late healing cannot be formally excluded in any individual case; however unlikely the prior clinical course makes it.

Consistent with the compassionate, observational nature of this report, batch-specific characterization of the secretome (protein and extracellular-vesicle content, growth-factor profile, and potency) and in vitro release or histological analyses were not performed here; the composition and bioactivity of this secretome preparation are characterized in the foundational studies [11,34].

Because the preparation was research-grade and was not released against compendial specifications, parameters such as bioburden, endotoxin content and defined potency were not formally tested; residual endotoxin, if present, could in principle influence the local inflammatory response. Future controlled studies should apply predefined release specifications including sterility, endotoxin limits, and a quantitative potency assay to the finished product.

Wound measurement was performed with a disposable ruler at clinical visits rather than by digital image-based measurement of the wound area, and closure was determined by the treating physician’s assessment rather than by assessment from a blinded independent observer; both introduce measurement imprecision [35]. Although HbA1c and ABI were available for Case 2, objective vascular indices and glycemic control data were not consistently recorded across all cases, limiting full characterization of the wound environment. Follow-up after closure was limited but confirmed durability in both cases: Case 1 remained healed at reviews on 31 January and 27 March 2026, and Case 2 at review on 21 June 2026 (approximately five months after closure); durability beyond these points is not established.

Finally, the preparation used was of research grade. Release testing consisted of a 72 h in-house microbiological screen with a negative control; no positive growth control was included, and no compendial sterility or bacterial endotoxin testing was performed. This is an acknowledged limitation of the material as used in these compassionate cases and would require replacement by validated, compendial release testing before any wider or regulated clinical use.

### 4.4. Implications

Within these constraints, the observations are encouraging. Two wounds that had defeated the available standard of care closed completely, in patients for whom the realistic alternatives were indefinite wound maintenance or, in the second case, further tissue loss. The preparation was well tolerated, with no local reactions in either patient. Case 2’s course was complicated by a wound infection in the second month, a common event in diabetic foot ulcers [5], not considered related to the preparation and which resolved after a brief interruption of treatment, following which healing continued to completion. No other adverse events occurred. This tolerability is consistent with the safety profile expected of an acellular, allogeneic-source material [22,36]. What these cases justify is not a claim of efficacy but a case for proper evaluation: a prospective, controlled study in patients with long-standing, treatment-refractory wounds, using complete closure as the primary endpoint, objective wound-area measurement, and a fully characterized preparation manufactured under validated conditions. The population that stands to gain most is the one currently least studied, this being older patients with vascular disease and wounds that everything else has failed to heal.

A prospective, controlled study is warranted, ideally incorporating comparator arms (standard care alone, carrier alone, and secretome-plus-carrier) with blinded outcome assessment. Complete closure should serve as the primary endpoint, with time-to-closure and planimetric percentage area reduction as secondary measures, supported by standardized serial photography using a fixed-scale marker and defined lighting and distance, and by follow-up of sufficient duration to capture recurrence.

## 5. Conclusions

Adjunctive treatment with a cell-free Wharton’s jelly-derived mesenchymal stromal cell secretome delivered in a thermoresponsive polymeric carrier resulted in complete healing of two chronic wounds: a two-year ulcer in a 95-year-old woman with peripheral arterial disease and a one-year neuro-ischemic diabetic foot ulcer after amputation and repeated debridement. Both wounds progressed to full re-epithelialization with modest total volumes of the preparation and good overall tolerability, the only intercurrent complication being a wound infection in Case 2 that resolved with a brief interruption of treatment.

A two-patient retrospective series cannot establish efficacy, and we make no such claim. What these cases do provide is a clinically grounded rationale for formal evaluation: they demonstrate that complete closure, rather than partial area reduction, is achievable in wounds that had already defeated the available standard of care, in patients whose age and vascular status place them outside the populations typically enrolled in trials of advanced wound therapies. Beyond the clinical observations, the intervention illustrates an engineered, cell-free delivery format. As a class, cell-free secretome preparations offer potential practical advantages over living-cell therapies, including amenability to storage and standardization and, being cell-free, freedom from the tumorigenicity and ectopic-differentiation risks associated with living-cell therapies [8,9]. The preparation used here was supplied with a manufacturer-defined refrigerated shelf-life established through the supplier’s stability testing. Cell-free secretomes are also, in principle, simpler and more scalable to produce than purified EV or exosome fractions, and here, the secretome was retained at the wound bed by a poloxamer-based thermoresponsive carrier that addresses the retention problem limiting liquid secretome preparations. These observations provide preliminary clinical justification for further controlled investigation of this combination in settings where refractory wounds are managed. Prospective, controlled study in this refractory population, using complete closure as the primary endpoint, objective wound-area measurement, and a preparation manufactured and released under validated conditions, is warranted.

## Figures and Tables

**Table 1:** Patient characteristics, treatment parameters, and outcomes.

	Case 1	Case 2
Age/sex	95/F	69/M
Comorbidities	Peripheral arterial disease (monophasic pedal Doppler)	Type 2 diabetes mellitus with neuropathy; peripheral arterial disease
Wound type	Chronic ankle ulcer (post-traumatic/infective, non-healing in the context of PAD)	Neuro-ischemic diabetic foot ulcer
Location	Anterior aspect, left ankle	Plantar aspect of forefoot
Duration prior to treatment	~2 years	~1 year
Prior treatments	Debridement; topical oxygen; Granulox; protease-modulating dressing; Endoform; Kerecis	Amputation (2nd ray, 3rd toe); surgical debridement; long-term IV antibiotics; Granulox; protease-modulating collagen dressing with Allevyn Ag
Maximal recorded size (cm)	3.8 × 4.0 × 0.2	3.0 × 6.5 × 0.7
Baseline size at treatment start (cm)	2.5 × 3.0 × 0.2	2.0 × 6.0 × 0.3
Baseline wound bed	70% fibrotic/30% granulating; rolled border; hyperpigmented periwound skin	100% granulation; ~1 cm undermining (reduced from ~2 cm)
Baseline wound area	~7.5 cm^2^	~12 cm^2^
Treatment start	3 July 2025	4 July 2025
Application frequency	Every 3 days	Every 3 days (reduced to weekly in final month)
Total volume applied	4 × 5 mL syringes (~20 mL)	4 × 5 mL syringes (~20 mL)
Adjunctive care	Weekly debridement for 3 months, then bi-weekly; periwound skin barrier	Bi-weekly debridement; PHMB cleansing; intermittent felt offloading; UrgoTul contact layer; Allevyn foam
Date of complete closure	8 January 2026 (sustained at 31 January and 27 March 2026)	18 January 2026 (sustained at 21 June 2026)
Time to complete closure	27 weeks	28 weeks
Adverse events	None	Wound infection in Month 2 (treatment paused ~3 weeks, then resumed); no other adverse events

## Data Availability

The data supporting the findings of this study are contained within the article. De-identified clinical records are available from the corresponding author on reasonable request, subject to patient confidentiality.
